# The impact of dementia on hospital outcomes for elderly patients with sepsis: A population-based study

**DOI:** 10.1371/journal.pone.0212196

**Published:** 2019-02-19

**Authors:** Carmen Bouza, Gonzalo Martínez-Alés, Teresa López-Cuadrado

**Affiliations:** 1 Health Technology Assessment Agency, Carlos III Health Institute, Madrid, Spain; 2 Department of Psychiatry, La Paz University Hospital, Madrid, Spain; 3 School of Medicine, Autonomous University of Madrid, Madrid, Spain; 4 National Epidemiology Centre, Carlos III Health Institute, Madrid, Spain; Azienda Ospedaliero Universitaria Careggi, ITALY

## Abstract

**Background:**

Prior studies have suggested that dementia adversely influences clinical outcomes and increases resource utilization in patients hospitalized for acute diseases. However, there is limited population-data information on the impact of dementia among elderly hospitalized patients with sepsis.

**Methods:**

From the 2009–2011 National Hospital Discharge Database we identified hospitalizations in adults aged ≥65 years. Using ICD9-CM codes, we selected sepsis cases, divided them into two cohorts (with and without dementia) and compared both groups with respect to organ dysfunction, in-hospital mortality and the use of hospital resources. We estimated the impact of dementia on these primary endpoints through multivariate regression models.

**Results:**

Of the 148 293 episodes of sepsis identified, 16 829 (11.3%) had diagnoses of dementia. Compared to their dementia-free counterparts, they were more predominantly female and older, had a lower burden of comorbidities and were more frequently admitted due to a principal diagnosis of sepsis. The dementia cohort showed a lower risk of organ dysfunction (adjusted OR: 0.84, 95% Confidence Interval [CI]: 0.81, 0.87) but higher in-hospital mortality (adjusted OR: 1.32, 95% [CI]: 1.27, 1.37). The impact of dementia on mortality was higher in the cases of younger age, without comorbidities and without organ dysfunction. The cases with dementia also had a lower length of stay (-3.87 days, 95% [CI]: -4.21, -3.54) and lower mean hospital costs (-3040€, 95% [CI]: -3279, -2800).

**Conclusions:**

This nationwide population-based study shows that dementia is present in a substantial proportion of adults ≥65s hospitalized with sepsis, and while the condition does seem to come with a lower risk of organ dysfunction, it exerts a negative influence on in-hospital mortality and acts as an independent mortality predictor. Furthermore, it is significantly associated with shorter length of stay and lower hospital costs.

## Introduction

Dementia is a global public health matter that affects 44 million people worldwide and costs 1% of global GDP [1]. It is estimated that the number affected will double in the next few years as a result of an ageing population and the lack of effective available options for prevention [2, 3]. Previous studies have shown that the prevalence of dementia among hospitalized patients is high [4–8], and on the rise [5,6]. In addition, various studies have highlighted that dementia increases the risk of death in elderly patients hospitalized for acute diseases [5, 6, 9] as well as hospital stays and costs [4, 6, 7, 10].

A large proportion of hospitalizations of patients with dementia are related with infectious processes and sepsis [5, 6, 8, 9, 11, 12]. In fact, sepsis is increasing in the elderly [13] and it is recognised as a global health priority [14] due to its high morbidity, mortality and utilization of hospital resources, especially when it presents with organ dysfunction [15]. Nevertheless, there is limited population-data information on the impact of dementia among elderly hospitalized patients with sepsis. Recently, a retrospective population-based study carried out in Taiwan [16] found that dementia increased the risk of organ failure and therefore mortality in hospitalized older adults. However, this study did not specifically analyse the characteristics or outcomes of the patients with sepsis, and for that reason it is difficult to understand the impact of dementia on the outcomes in elderly hospitalized patients with sepsis.

Spain is one of the countries with the highest prevalence of dementia worldwide [3] and this study aims to analyse the epidemiological characteristics and the impact of dementia on organ failure, in-hospital mortality and the utilization of hospital resources by adults aged ≥65years with sepsis via the Spanish national database of hospital discharges. Our hypothesis is that patients with dementia have a higher risk of acute organ dysfunction, greater rates of in-hospital mortality and use more hospital resources.

## Materials and methods

### Design and data source

The data used come from the official database (CMBD) of the Spanish National Health System (Ministry of Health, Social Services and Equality). According to the regulations of the Spanish National Health System, every health professional should enter–at the moment of each patient’s hospital discharge–all of the diagnoses and procedures carried out, using the 9^th^ revision of the International Classification of Diseases (ICD-9-CM) and the associated “diagnosis-related groups” or DRGs. This information, the completion of which is a legal requirement, is gathered together in a national database called the “Minimum Basic Data Set” (*Conjunto Mínimo Básico de Datos*, CMBD), which includes more than 97% of the hospital discharges that occur in Spain each year, and is considered representative of the national population [17]. In the CMBD each hospitalization is treated as a specific record, and includes demographic information, type of admission, dates of admission and discharge, destination on discharge, primary diagnosis, 13 secondary diagnoses and up to 20 procedures carried out during the hospitalization [17–18].

### Population studied. Identification of cases and definitions

We included hospitalizations of ≥ 65s with sepsis from January 1^st^ 2009 to 31 December 2011. In order to identify sepsis, previously used codes to define infection were employed [19–21]: 038 (038.0, streptococcal septicaemia; 038.1, staphylococcal septicaemia; 038.2, pneumococcal septicaemia; 038.3, septicaemia due to anaerobes; 038.4, septicaemia due to other Gram-negative organisms; 038.8, other specified septicaemias; 038.9, unspecified septicaemia); 003.1 (*Salmonella* septicaemia); 020.2 (septicaemic plague); 036.2 (meningococcal septicaemia); 036.3 (Waterhouse–Friderichsen syndrome); 054.5 (herpetic septicaemia); 098.89 (gonococcaemia); 112.5 (systemic candidiasis); 112.81 (candida endocarditis); 117.9 (other and unspecified mycoses); and 790.7 (bacteraemia). The ICD-9-CM code for sepsis, 995.91 (sepsis, systemic inflammatory response syndrome due to infectious process without organ dysfunction), which was introduced in Spain in January 2004, was also included [17].

To identify cases with acute organ dysfunction, we used ICD-9-CM code 995.92 (severe sepsis, sepsis with organ dysfunction)–as well as the codes that specifically define organ dysfunction. These specific codes were [19,20]: respiratory: 518.81 (acute respiratory failure), 518.82 (other pulmonary insufficiency), 518.84 (acute on chronic respiratory failure), 518.85 (acute respiratory distress syndrome after shock or trauma), 786.09 (respiratory distress, insufficiency), 799.1 (respiratory arrest), 96.7 with all sub-codes (invasive mechanical ventilation); cardiovascular: 785.5 with all sub-codes (shock without mention of trauma, includes 785.51, 785.52, 785.59), 458 (hypotension, 458.0, 458.8 458.9), 796.3 (nonspecific low blood pressure reading); renal: 584 with all sub-codes (acute renal failure), 580 (acute glomerulonephritis), 39.95 (haemodialysis); hepatic: 570 (acute and subacute necrosis of liver), 572.2 (hepatic coma), 573.3 (hepatitis, unspecified); hematologic: 286.6 (defibrination syndrome), 286.9 (other and unspecified coagulation defects), 287.3–5 (secondary thrombocytopenia, unspecified); neurologic: 293 (Transient organic psychotic conditions), 348.1 (anoxic brain damage), 348.3 (encephalopathy, unspecified), 357.82 (critical illness polyneuropathy), 780.01 (coma), 780.09 (drowsiness, unconsciousness, stupor), 89.14 (electroencephalogram) and metabolic: 276.2 (acidosis, metabolic or lactic).

This combination of codes from the ICD-9CM has shown capable of accurately estimating the burden of sepsis and organ dysfunction [21], and has been used previously by our research group [22]. Dementia cases were identified by the presence, in the principal or secondary diagnoses, of the following ICD-9CM codes: 290 (dementias), 294.1 (dementia in conditions classified elsewhere), 294.2 (dementia, unspecified), 331.0 (Alzheimer’s disease), 331.1 (frontotemporal dementia), 331.2 (senile degeneration of brain), 331.82 (dementia with Lewy bodies) [4,7]. The cases with and without dementia were compared in relation to the primary variables of interest–including the presence of organ failure, in-hospital mortality, and hospital resource utilization (length-of-stay and cost). These variables were also examined according to age, given its influence on patients’ outcomes and the use of resources in septic patients [13]. Likewise, we analysed other demographic characteristics, such as sex or institutionalization status (whether the subject lived in a nursing home before hospitalization), as well as other covariates of clinical importance like the burden of comorbidities, the potential site of infection, microbiological data and whether invasive life-support procedures were used. These procedures were defined as: infusion of vasopressor agent (ICD-9CM code: 00.17); continuous invasive mechanical ventilation (ICD-9CM code: 96.70, 71 y 72); and haemodialysis (ICD-9CM: 39.95). In order to explore comorbidity, we used the Charlson Index in the version validated by Deyo for use on administrative databases [23] and improved for ICD-9CM [24] for the 14 diagnosis fields. This index includes specific comorbid conditions of known prognostic value, which are classified using ICD-9 codes from prior outpatient and inpatient codes. Previous epidemiological studies have shown its usefulness in assessing risk of death in septic patients [25]. For the purposes of this study, the presence of dementia in the Charlson Index was excluded [12, 16]. To be able to identify specific microorganisms, we used code 041, which, according to the ICD-9-CM coding manual, is used as an additional code to identify the bacterial agent in diseases classified elsewhere [17].

### Ethics

The data are anonymized and, according to Spanish law, are exempt from the necessity for informed consent [26]. They come from hospital discharge records collected and de-identified by the Spanish Ministry of Health, Social Services and Equality. The authors requested and obtained access to the data from the Ministry and, due to a signed confidential agreement under the project PI09/0597, cannot share these data with third parties. However, these records are publicly available for research purposes. Requests of access to the data should be addressed directly to the Ministry [27].

### Analysis of data

We carried out descriptive and comparative analyses of the cases with and without dementia, including clinical and demographic data, the burden of comorbidities, the presence of organ failure, in-hospital mortality, length-of-stay and hospital costs. The Charlson Index was calculated–using the improved version of Stata 14 –and expressed as a continuous variable and, in addition, as a category in 4 groups (0, 1–2, 3–4, >4) of increasing severity and impact on outcomes [28]. Case Fatality Rate (CFR) was calculated as the number of deaths divided by the number of cases and expressed as a percentage. The quantitative variables are presented as means with standard deviations, and the categories as overall counts and percentages. The association between qualitative variables was analyzed via the Pearson χ^2^ test or Fisher’s exact test. A t-test was used to compare continuous variables.

The specific effect of dementia on the primary endpoints was calculated using multivariate regression models. In order to evaluate the effect on organ dysfunction we used two different adjusted logistic regression models: Model 1 included the main baseline characteristics: sex, age and the burden of comorbidities, whereas Model 2 also included the identification of the pathogen and the site of infection.

Regarding in-hospital mortality, before carrying out the multivariate analysis, we performed an exploratory analysis, calculating the independent effect of dementia on each of the principal covariates (sex, age, comorbidities, identification of pathogens, site of infection, and presence of organ dysfunction). Following this, we used adjusted logistic-regression techniques on three models, which included Models 1 and 2 and an additional third model that included the presence of organ failure.

For the continuous variables relating to hospital resources (length-of-stay and costs), we employed multivariate linear-regression models, adjusted by two further models: Model 4, included baseline characteristics, identification of pathogen and site of infection, and presence of organ dysfunction; Model 5 added invasive therapeutic measures to the variables in Model 4.

The results of logistic regression models are presented as odds ratios (ORs) with 95% confidence intervals (95%CI), and those of the linear regression models as coefficients (β), also with 95% confidence intervals. The statistical analysis was carried out using STATA 14 (1985–2015 StataCorp LP. TX 77845 USA). Results were considered significant with a p-value <0.05.

## Results

In the period analyzed, there were 148 293 entries with sepsis in adults aged ≥ 65 years, of which 16 829 cases (11.3%) had a diagnosis of dementia. Around 45.8% of cases (n = 7712) were coded as Alzheimer’s disease, 18.5% (n = 3119) as vascular dementia and the remaining 35.6% (n = 5998) was made up of mixed dementias and other types of dementia.

### Clinical and demographic characteristics

As shown in Table 1, the cohort with dementia showed a clear predominance of women and a greater mean age—more than half of the cases were 80 or over, while 54% of the cases without dementia were under that age. Although in both groups hospital admission was primarily non-elective through emergency departments, there were differences between groups, and planned admission was significantly lower in cases with dementia.

Before hospital admission, 11.9% of the cases with dementia were institutionalized compared to 3.1% of those without dementia.

**Table 1:** General characteristics of the population 2009–2011.

	With dementia	Without dementia	p-value
	16 829 (11.3)	131 464 (88.7)	
**Year of Study**			0.505
2009	5244 (11.3)	41 360 (88.7)	
2010	5551 (11.3)	43 556 (88.7)	
2011	6034 (11.5)	46 548 (88.5)	
**Sex**			<0.001
Women	9587 (57)	57 708 (43.9)	
**Age, years**			<0.001
65–69	379 (2.2)	18 624 (14.2)	
70–74	1007 (6.0)	22 205 (16.9)	
75–79	2811 (16.7)	30 734 (23.4)	
80–84	4860 (28.9)	28 248 (21.5)	
85–89	4920 (29.2)	20 592 (15.7)	
≥90	2852 (17.0)	11 061 (8.4)	
Mean±SD ^#^	83.6±6.3	78.7±7.7	<0.001
**Type of hospital admission**			<0.001
Non-elective	16 077 (95.5)	120 026 (91.3)	
**Institutionalized**	1995 (11.9)	4121 (3.1)	<0.001
**Medical pathology**	15 980 (95.0)	103 890 (79.0)	<0.001
**Sepsis as reason for admission**	10 391 (61.7)	56 778 (43.2)	<0.001
**Charlson Index**			<0.001
0 points	5991 (35.6)	28 506 (21.7)	
1–2 points	8330 (49.5)	61 098 (46.5)	
3–4 points	2100 (12.5)	27 986 (21.3)	
>4 points	408 (2.4)	13 874 (10.5)	
Mean±SD^#^	1.23±1.34	2.06±1.90	<0.001
**Principal comorbidities**			
Diabetes	4906 (29.2)	35 607 (27.1)	<0.001
Cerebrovascular disease	2657 (15.8)	11 739 (8.9)	<0.001
Chronic kidney disease	2167 (12.9)	23 121 (17.6)	<0.001
Congestive heart failure	1936 (11.5)	23 388 (17.8)	<0.001
Chronic obstructive pulmonary disease	1789 (10.6)	23 852 (18.1)	<0.001
Cancer	954 (5.7)	27 498 (20.9)	<0.001
Peripheral vascular disease	740 (4.4)	8270 (6.3)	<0.001
Acute myocardial infarction	471 (2.8)	5834 (4.4)	<0.001
**Site of infection**	11 248 (66.8)	94 362 (71.8)	
Genitourinary	6803 (40.4)	38 259 (29.1)	<0.001
Respiratory system	3023 (18)	22 761 (17.3)	0.036
Soft tissue	573 (3.4)	4923 (3.7)	0.028
Abdomen	359 (2.1)	9355 (7.1)	<0.001
**Identification of pathogens**	7160 (42.6)	71 081 (54.1)	<0.001
Gram-negative bacteria	5159 (72.0)	47 798 (67.2)	<0.001
Gram-positive bacteria	2591 (36.2)	28 660 (40.3)	<0.001
Number of organ dysfunction**			<0.001
None	6493 (38.6)	48 123 (36.6)	
1	5979 (35.5)	39 542 (30.1)	
≥2	3391 (20.2)	39 646 (30.2)	

Chi-squared Test

^#^ Student’s T-Test; SD: Standard Deviation

**in 3.4% of cases the number of organ failures was not specified.

Likewise, while the reasons for admission were primarily medical in both groups, the percentage of surgical cases was significantly lower in the cohort with dementia, with 5% of cases. Sepsis was the cause of admission for 62% of cases with dementia, and 43% of the cohort without dementia; being the differences statistically significant.

In Table 1, the values from the Charlson Index show that the cohort with dementia had a significantly lower burden of comorbidities than the cohort without dementia. In addition, except for diabetes and cerebrovascular diseases, the frequency of specific comorbidities was significantly lower in the cohort with dementia.

The most frequent potential sources of sepsis were genitourinary, being the percentage of cases significantly greater in the cohort with dementia (40% vs. 29%) and respiratory (18% vs. 17.3%). However, both the identification of pathogens (42.6% vs. 54.1%) and the presence of bacteraemia (18.2% vs. 29.4%) were significantly lower in the cohort with dementia. In both cohorts the most frequent pathogen identified was gram-negative bacteria.

### Organ dysfunction

As shown in Table 1, almost 40% of cases overall were not suffering from organ dysfunction with a slightly–but significantly–larger proportion being found in the cohort with dementia. Furthermore, the percentage of dementia cases who presented with a single dysfunction was greater (35.5% vs. 30.1%), while this relationship was inverted in those cases with dysfunction of two or more organs.

Similar differences can be observed in Table 2 for the specific dysfunctions analyzed, and the cohort with dementia showed a significantly lower frequency for each. In both cohorts respiratory dysfunction was the most common, followed by kidney dysfunction and then cardiovascular, whose occurrence was notably different between cohorts.

**Table 2:** Effects of dementia on acute organ dysfunction in patients with sepsis.

	With dementia Cases (%)	Without dementia Cases (%)	Model 1 OR (95%CI)	Model 2 OR (95% CI)
**Acute organ dysfunction**	10 336 (61.4)	83 341 (63.4)	0.93 (0.90, 0.97)	0.84 (0.81, 0.87)
Respiratory	4190 (24.9)	38 362 (29.2)	0.85 (0.82, 0.89)	0.80 (0.77, 0.83)
Cardiovascular	3329 (19.8)	37 089 (28.2)	0.67 (0.64, 0.70)	0.59 (0.57, 0.62)
Renal	3957 (23.5)	39 431 (30.0)	0.75 (0.72, 0.78)	0.71 (0.69, 0.75)
Hepatic	164 (1.0)	3411 (2.6)	0.64 (0.55, 0.76)	0.61 (0.52, 0.72)
Hematological	523 (3.1)	8804 (6.7)	0.48 (0.44, 0.53)	0.46 (0.42, 0.51)
Metabolic	645 (3.8)	7823 (6.0)	0.61 (0.56, 0.66)	0.56 (0.51, 0.61)
Neurological	1033 (6.1)	9137 (7.0)	0.85 (0.80, 0.91)	0.85 (0.79, 0.91)

Model 1: adjusted for sex, age and points on the Charlson Index

Model 2: adjusted for sex, age, points on the Charlson Index, identification of pathogens and site of infection

Table 2 also presents the results of the multivariate analysis in which the impact of dementia on the presence and type of organ dysfunction was analyzed. The first model was adjusted for age, sex and points on the Charlson Index; the second, additionally adjusted for the site of infection and identification of pathogens. After adjustment, in Model 1, dementia was associated with 7% lower risk of presenting with organ dysfunction than the cohort without dementia. Meanwhile, in Model 2, this difference increased to 16%. In the individual analyses of the individual types of dysfunction, in all of them and in both models, dementia was associated with a significantly lower risk.

Among cases who presented with organ dysfunction, 3.6% (n = 376) in the dementia cohort and 25.5% (n = 21 280) in the without dementia cohort received invasive therapeutic measures for organ-system support; the difference was statistically significant (p<0.001).

### Mortality

The CFR was 43% (n = 7276) in the cohort with dementia and 34% (n = 45 187) in the non-dementia cases, and these differences were statistically significant.

**Fig 1:**
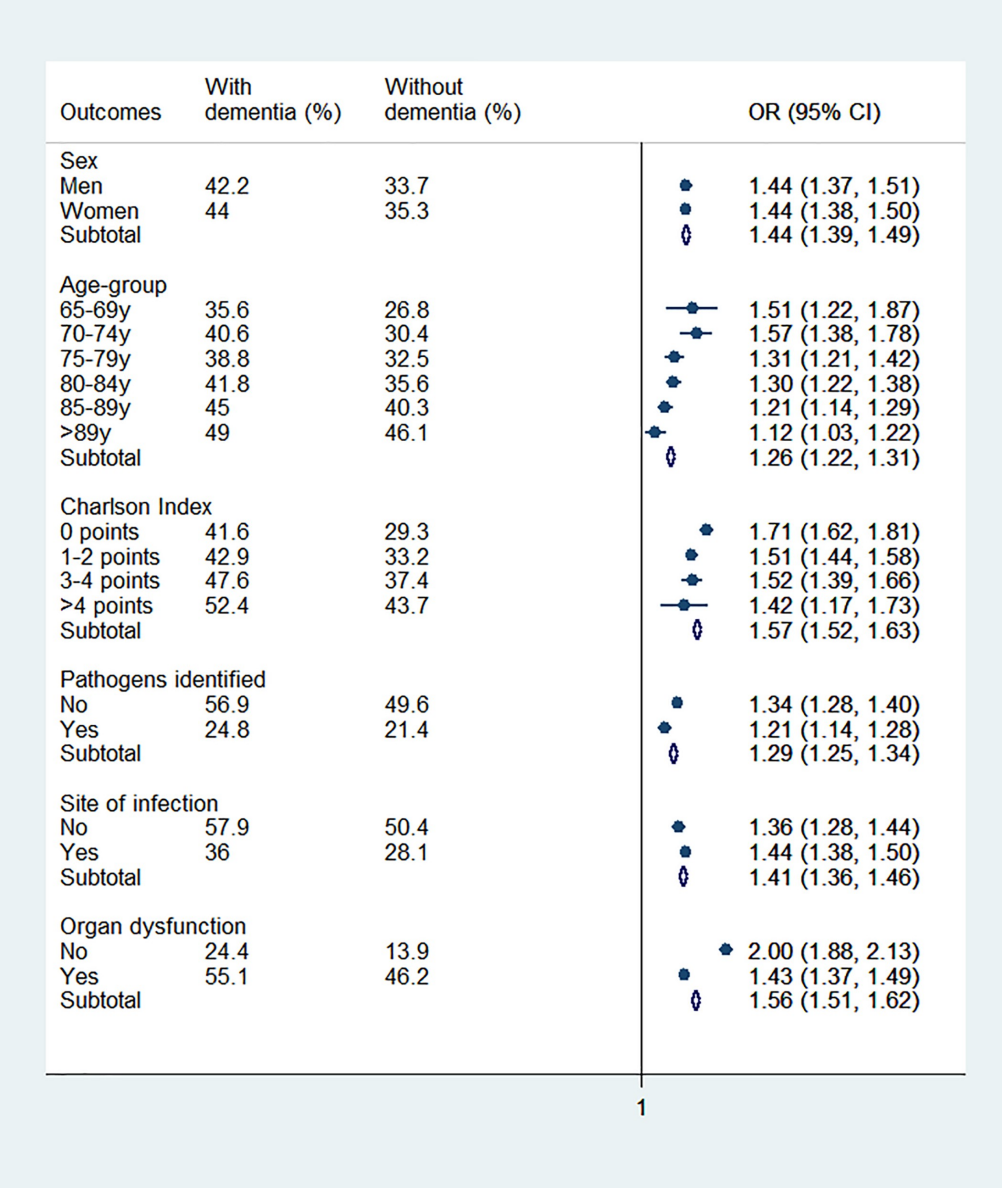
Likelihood of in-hospital mortality from principal covariates in dementia cases compared with non-dementia cases.

In both cohorts (see Fig 1), to be a woman, of more advanced age, with a greater burden of comorbidities, the non-identification of the site of infection or the pathogen, and the presence of organ dysfunction were all associated with higher mortality. Nevertheless, as it can be seen, there was a significant difference in mortality between cohorts, with higher values for the dementia cohort in all of the variables analyzed.

The same chart shows the effect of dementia on mortality, adjusted for each of the variables. These results indicate that dementia independently augments the mortality risk for each variable analyzed, while the extent of the effect is greater in specific groups, namely: the younger patients with less comorbidities and organ dysfunction. Thus, adjusting for age, the mortality of cases with dementia is 26% higher than cases without dementia. For subjects between 65 and 69 years old, the OR of death is 1.51 times higher in the patients with dementia, while it decreases to 1.12 times for those ≥90. The same occurs with the Charlson Index, where the impact of dementia on mortality shows an OR of 1.71 in cases without comorbidity and of 1.42 where there is a higher burden of comorbidities. Having analyzed organ dysfunction, the results of the analysis indicate that in cases without organ dysfunction the risk of death is twice as high in patients with dementia, while said risk is 43% higher in cases with organ dysfunction.

As regards the utilization of therapeutic measures for organ-system support, although they are used in 86% fewer cases in the cohort with dementia. In those cases where these measures are employed, we did not observe significant differences in the mortality of the two groups (OR:0.89, 0.72–1.09).

In Table 3, the results of the multivariate regression analysis show the impact of dementia on in-hospital mortality. The cohort with dementia presents a risk of death 36% higher than the cohort without dementia, controlled for baseline covariables (Model 1). Nevertheless, this risk is reduced to 23% if the detection of pathogens and the potential site of infection are controlled for (Model 2), and to 32% when the presence of organ dysfunction is also controlled for.

**Table 3:** Impact of dementia on in-hospital mortality.

	With dementia (%)	Without dementia (%)	Adjusted OR (95%CI)		
			Model 1	Model 2	Model 3
**Mortality**	43.2%	34.4%	1.36 (1.32, 1.41)	1.23 (1.19, 1.27)	1.32 (1.27, 1.37)

Model 1: adjusted for sex, age and points on the Charlson Index

Model 2: adjusted for sex, age, points on the Charlson Index, identification of pathogens and site of infection

Model 3: adjusted for sex, age, points on the Charlson Index, identification of pathogens and site of infection and presence of organ dysfunction

### Utilization of resources

**Fig 2:**
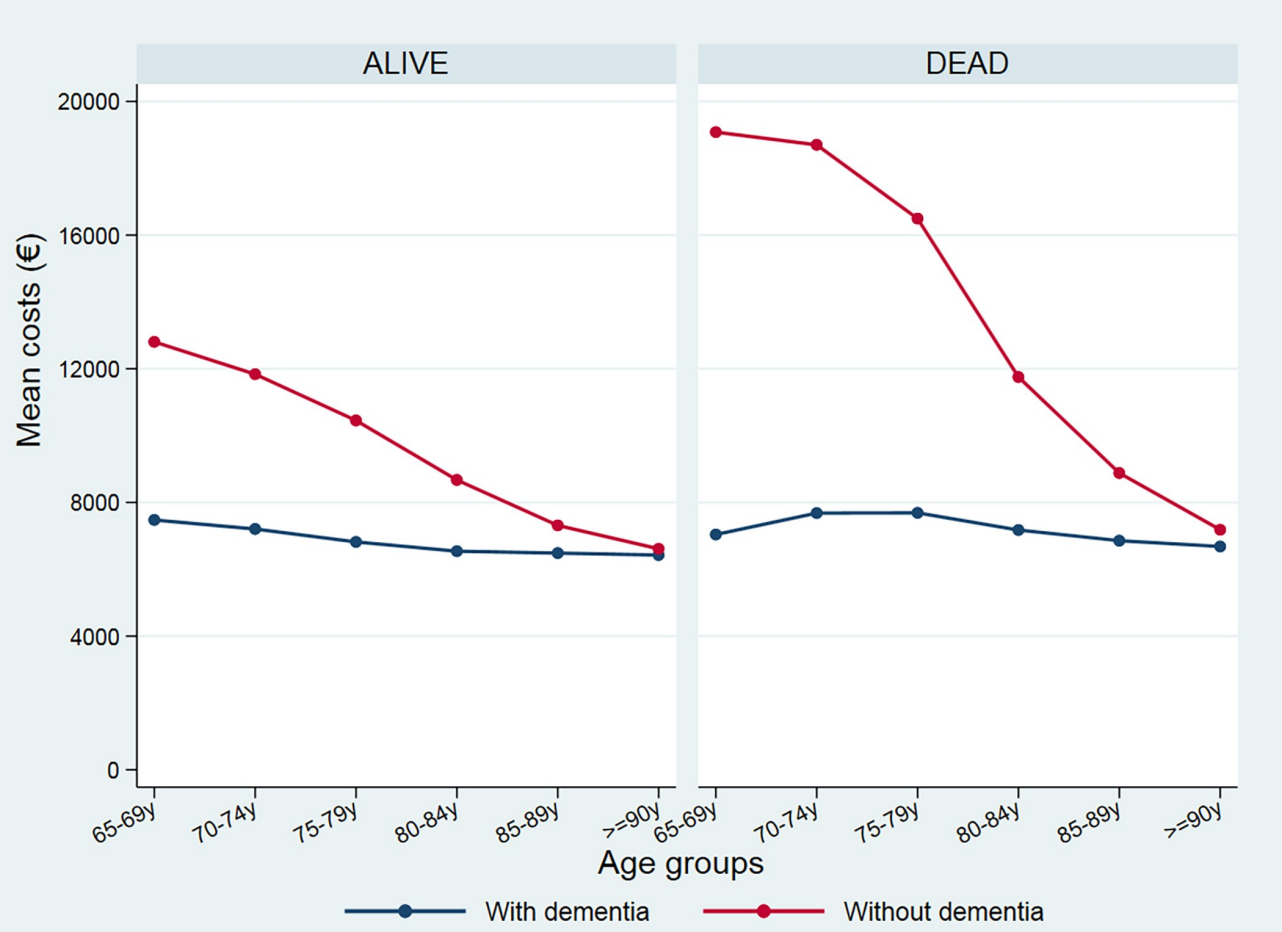
Mean length-of-stay for patients with and without dementia by age and vital status on discharge.

Both the average length-of-stay and the mean hospital costs were significantly lower in the cohort with dementia. The mean stay was 11.3 days for the cohort with dementia and 17.4 days for the without dementia cases. These differences are maintained when analyzing the cases who died as well as those who survived their stay. For cases who died the mean stay was 8.6 days in the cohort with dementia, against 14.7 days in the cohort without dementia. As shown in Fig 2, the mean length-of-stay decreases with increased age, but the decline is clearly less pronounced in the cohort without dementia and the differences between both groups are much more marked between 65 and 85 years of age.

**Fig 3:**
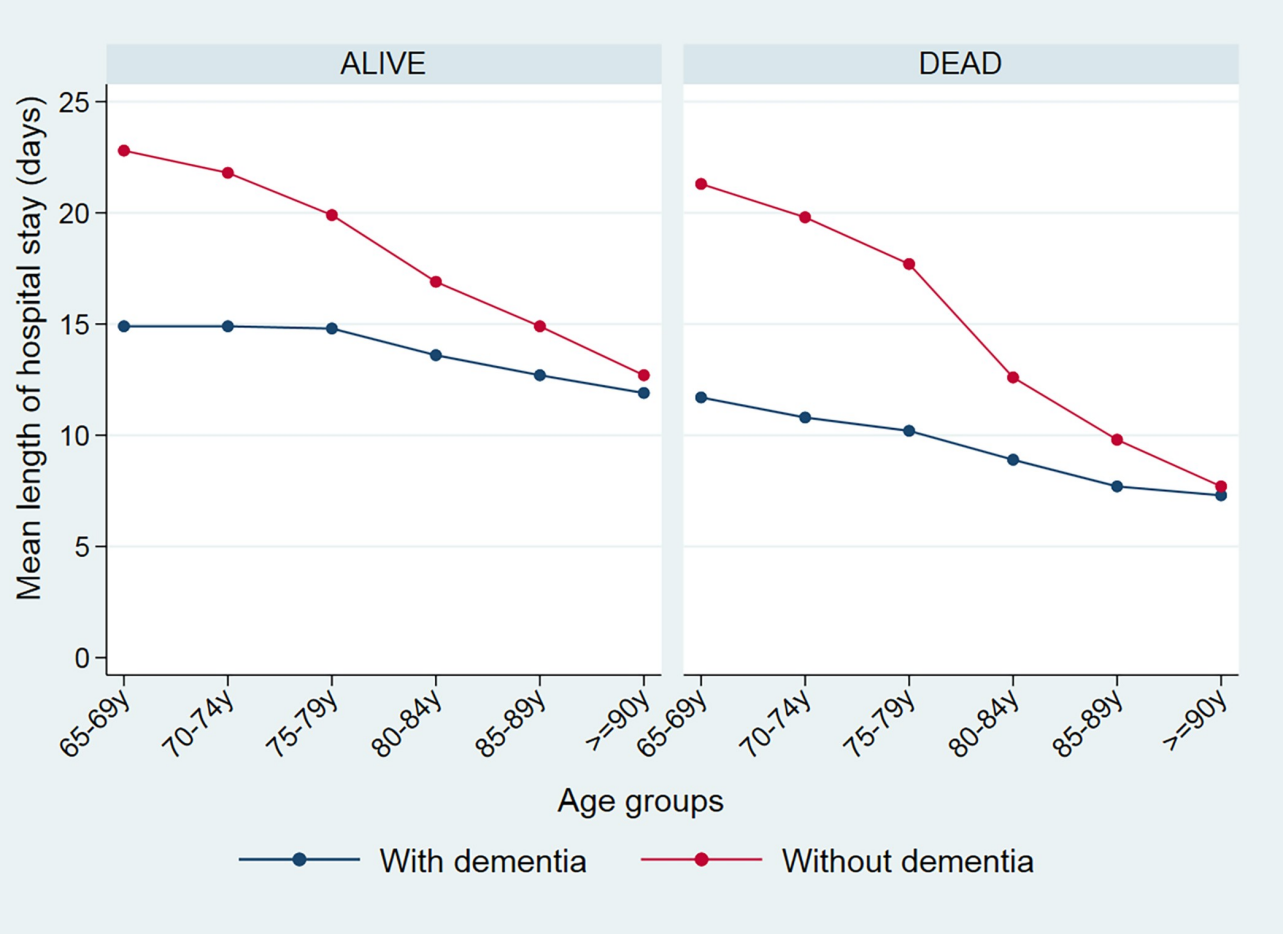
Mean hospital costs for patients with and without dementia by age and vital status on discharge.

As regards the costs, Table 4 shows a mean hospitalization cost of €6824 in the cohort with dementia and €11,230 for cases without dementia. Fig 3 shows the mean costs for both cohorts, as related to age and status on discharge; in the same figure we can see that–for the without dementia cases–the mean costs are much greater in cases where the patient died than in those that survived their hospital stay, and that in both cases the figure falls with increased age. In cases with dementia the cost is practically stable for both living and dead in the different age ranges. The curves of both cohorts show a substantial difference between 65 and 80 years of age, and tend to overlap from 90 onwards.

**Table 4:** Impact of dementia on utilization of hospital resources.

	With dementia	Without dementia	Adjusted (95%CI)	
Hospital Resources			Model 4	Model 5
Length-of-stay, mean number of days	11.3±12.4	17.4±21.4	-3.87 (-4.21,-3.54)	-2.69 (-3.02, -2.36)
Costs, € mean	6824±4260	11 230±15 793	-3040 (-3279, -2800)	-1489 (-1713, -1266)

Model 4: adjusted for sex, age, Charlson Index, and presence of organ dysfunction

Model 5: adjusted for sex, age, Charlson Index, presence of organ dysfunction, and invasive therapeutic measures

The results of the multivariate linear regression model employed to evaluate the impact of dementia on the utilization of hospital resources is shown in Table 4. This analysis, adjusted for age, sex, points on the Charlson Index and the presence or non-presence of organ dysfunction, indicates that dementia involves a 3.87-day reduction in length of hospital stay. This difference is reduced to -2.69 days if adjustment for the use of invasive therapeutic measures is accounted for. The difference in mean hospital costs per case is €3040 lower (95%CI: -3279, -2800) in patients with dementia, but this is reduced to -1489 euros (95%CI: -1713, -1266) when invasive therapeutic measures for life support are incorporated into the model.

## Discussion

This nationwide population-based study shows that dementia is present in a substantial proportion of adults ≥65s hospitalized with sepsis, and while the condition associates with a lower risk of organ dysfunction, it exerts a negative impact on in-hospital mortality and acts as an independent mortality predictor. The negative impact of dementia on mortality is greater in lower age-groups, groups with a lower comorbidity burden and in cases without organ dysfunction. Furthermore, it is significantly associated with shorter length of stay and lower hospital costs.

As far as we know, this is the first population-level study which investigates the epidemiology and outcomes of hospitalized patients with sepsis and dementia. Our data shows that dementia is common in the adult population ≥65 with sepsis–affecting 11.3%, with a distribution that increases progressively with age and that more than half of the cases are women. These findings are in line with the literature, although the observed frequency is somewhat higher than that referred to in studies which analyze its prevalence in the general hospital population [6, 7, 12, 29, 30]. Also, Alzheimer’s disease is common and represents almost half of the cases with dementia [29, 31–33]. It is important to highlight that in 62% of cases with dementia, sepsis was the reason for hospital admission. This figure, significantly higher than that for cases without dementia, suggests a community-acquired origin of sepsis in a large proportion of those patients. In line with literature, the most frequent site of infection was urinary followed by respiratory [4, 5, 8, 12].

Several studies have demonstrated that mortality from sepsis is associated with greater age, greater burden of comorbidities and, in particular, with the presence of organ dysfunction [13, 15, 19–22]. The results of our study accord with these previous studies, in addition to showing that the existence of dementia is an independent factor associated with higher mortality in this population.

In this study, the cohort with dementia presents a lower burden of comorbidity as evaluated through the Charlson Index, which does in fact contrast with some previous publications [7, 12, 34], but agrees with others [6, 30, 33]. Although we cannot discount that patients with dementia are subject to under-diagnosis of comorbidities, the presence of a profile with higher diabetes and cerebrovascular-disease prevalence agrees with the results of prior studies [6, 30, 35, 36] and suggests that the coding is not systematically biased.

Moreover, the cases with dementia showed a significantly lower risk of acute organ dysfunction than those without dementia, after adjusting–in the multivariate analysis–for demographic and clinical characteristics known as predictive factors for outcome such as age, sex, burden of comorbidity, the identification of a potential site of infection and the responsible microorganism. Our findings contrast with those described by Shen et al. in Taiwan [16] where dementia was associated with a higher risk of organ dysfunction. This discrepancy might be due to methodological and design differences between the two studies, as their population consisted of ≥65years hospitalized for any cause and they did not specifically analyze cases of sepsis. This great difference and the lack of information about cases with sepsis make it impossible to adequately compare the results. That said, we have confidence in the results of our study given that it uses cases identified through well-used and validated strategies for the epidemiological analysis of sepsis and its outcomes [13,19–22]. In addition, as the study is based upon population data whose declaration is obligatory and not subject to systematic selection bias, there is no reason to presume that the registration of organ dysfunctions would be different between cohorts. By the same token, given the universal character of our national health system we can assume that coding practices were not due to economic incentives. Unfortunately, we have not identified any other studies that analyze the outcomes of acute organ dysfunction in patients with dementia and sepsis with which we might compare our data.

In relation to age, for patients with dementia and sepsis the increased risk of death with greater age is clearly lower than in the without dementia cases. In addition, although cases with dementia are, on average, around five years older than those without dementia, the greatest differences in mortality between the cohorts are found among lower age ranges. Along similar lines, the impact of dementia on mortality has a greater effect on clinically less severe groups–with fewer comorbidities and without organ dysfunction. Regrettably, the design of this study does not permit us to identify the causes of these differences, but our results suggest that dementia is a mortality risk factor whose impact is less perceptible with more advanced ages and/or greater clinical severity [16, 37].

The presence of dementia has a negative association with indicators of clinical management, like the identification of the site of infection, the pathogen responsible for causing the sepsis or the use of invasive techniques for organ support. The correct identification of the site of infection and the responsible microorganism are critical for clinical management of sepsis, as the administration of ineffective antibiotic increases mortality [15, 38, 39]. It must be acknowledged that the clinical manifestations of sepsis are variable and may be subtle in elderly patients–and that dementia presents additional challenges due to communication difficulties and poor tolerance for diagnostic procedures and other care activities. Nonetheless, the implementation of educational programmes for professionals on the diagnosis and early treatment of sepsis has improved patients outcomes and reduced mortality [40, 41]. Along the same lines, the results of this study suggest that it is necessary to undertake greater diagnostic efforts in these patients–once the identification of the potential site of infection and pathogen responsible is accomplished, the impact of dementia on in-hospital mortality should shrink markedly.

It is also important to mention that the use of withdrawing or withholding invasive life support measures may provide a partial explanation to some of our findings. The use of invasive therapeutic measures in patients with dementia is under debate–as the appropriateness of their use and their results are far from clear [42–44] and, as stated by Richardson [42], acute care patients with dementia are treated substantially less aggressively than patients without dementia. Conversely, a recent study by Lagu in the US, however, shows that between 2001 and 2011 the utilization of invasive mechanical ventilation increased four times more in patients with dementia than in those without dementia [45]. In our study these measures were used in a small percentage of cases with dementia, but in those cases where they were used we did not observe significant differences in mortality between the two groups. This suggests that the selection criteria for the use of these measures were evaluated from the perspective of appropriate care and concurs with the findings of recent publications [16, 43, 46]. Unfortunately, our study design rendered it impossible to assess cases in which there have been decisions to forgo life-sustaining measures and we are unaware of the influence of patients’ advanced directives in our study population. Further, studies investigating the frequency and quality of palliative care in critically ill patients with dementia are scarce [47]. However, the results of previous studies in which we participated show that, in our country, these decisions are taken by professionals on the basis of biomedical reasons of therapeutic futility or ineffectiveness, that their attitude tends to be conservative and less pro-active than in other countries, and that such decisions are taken in approximately 6.6%-9.8% of patients admitted to the Intensive Care Unit [48,49]. Furthermore, besides age and acute and chronic diagnoses, quality of life and functional status have the greatest impact on decisions to limit life support [48]. Accordingly, it seems eminently plausible that the presence and severity of dementia may determine less aggressive clinical management decisions and patients with dementia and sepsis may be less likely to be treated with invasive measures.

Previous studies have noted that dementia increased the mean length-of-stay and the costs of hospitalization for elderly patients diagnosed with acute conditions [5, 10]. However, our study provides contrasting results: length-of-stay and hospital costs were significantly lower for patients with dementia, both overall and when examining the cases who died or survived their hospital stay. Overall, the adjusted differences observed in our study go up to almost four days hospital stay and more than €3000 mean hospitalization costs. The lower rates of organ dysfunction and the lower use of life-support invasive measures in the dementia cohort as compared to dementia-free cases, may explain these findings. Additionally, even if in the multivariate model the adjusted size of the difference between the two groups–for both length-of-stay and costs incurred–was reduced when the use of invasive therapeutic measures was introduced as a covariate, notable differences remained. Interestingly, Lagu et al. [46], in their recent retrospective population-study of 65s and over, found that patients with dementia under invasive mechanical ventilation had shorter lengths-of-stay and lower hospital costs than those without dementia.

Our data also show that the differences in these estimators between the two cohorts are greater at lower ages. This is possibly because the gap between clinical attitudes for subjects with and without dementia, regarding the use of life-support invasive measures tends to narrow with the advancing age of patients–with an overall reduction in therapeutic efforts [42, 43, 46,48,49].

Our results expand the still scarce information available about the impact of dementia on elderly patients hospitalized with sepsis. This study used the nationwide official database of hospital discharges of the Spanish National Health System. Data entry into this database is mandatory by law, covers over 97 per cent of the all acute-care, public and private, hospitalizations nationwide and is subjected to regular audits to verify the adequacy and accuracy of the coding used [17–18]. Thus, we consider that our results could be generalized. These results could be of interest for decision-makers and the planning of care resources in an increasingly ageing society for which a greater prevalence of dementia and sepsis is foreseen in the near future.

The limitations of our study are those inherent to investigations based on retrospectively collected clinical-administrative data. Although there are national directives for the use of the ICD-9-CM coding system, this may not have been uniform across all hospitals of the national health network and we cannot rule out coding errors despite regular audits making major errors unlikely. However, we have limited the study to a spread of recent years, as it is known that the coding in Spain has improved over the last few years. A further limitation is the absence of diagnostic information about the severity of dementia [50, 51]. Accordingly, it is necessary to bear in mind that all of these are hospital cases, and it has already been demonstrated that there is an inverse relation between the severity of the dementia and the probability of being hospitalized [52]. Moreover, we lack data about other socio-demographic characteristics like risky behaviour; pharmacological treatments; economic, cultural or educational level. This impeded our ability to better characterize the pattern of this cohort and stratify the results by severity of dementia and other risk factors. Nonetheless, the inclusion of cases which covered all subjects at the population level allows us to assume that none of the groups compared were selected with systematic bias.

Lastly, our analysis does not include non-hospitalized cases. Thus, the mortality data is in-hospital mortality and prior studies have demonstrated that patients who survive a septic episode have a higher risk of death during the following months or even years [15, 53] which makes the estimates conservative.

In conclusion, this study shows that dementia is present in a significant proportion of ≥65 patients hospitalized with sepsis, and that while the condition does not seem to come with a higher risk of organ dysfunction, it both exerts a negative impact on hospital mortality and acts as an independent mortality predictor. According to our results this impact is higher in lower age-groups, groups with a lower comorbidity burden and in cases without organ dysfunction. In addition, it is associated with a significantly lower length of stay and hospital cost. Further study is needed into the factors associated with the negative effect of dementia in elderly patients with sepsis.
